# Polyphenols from the extract and fraction of *T. indica* seeds protected HepG2 cells against oxidative stress

**DOI:** 10.1186/s12906-015-0963-2

**Published:** 2015-12-18

**Authors:** Nurhanani Razali, Sarni Mat Junit, Azhar Ariffin, Nur Siti Fatimah Ramli, Azlina Abdul Aziz

**Affiliations:** Department of Molecular Medicine, Faculty of Medicine, University of Malaya, 50603 Kuala Lumpur, Malaysia; Department of Chemistry, Faculty of Science, University of Malaya, 50603 Kuala Lumpur, Malaysia

**Keywords:** *Tamarindus indica* seeds, antioxidant, MDA, 4-HNE, flavonoids, polyphenols, UHPLC, NMR, HepG2 cells

## Abstract

**Background:**

*Tamarindus indica* L. (*T. indica*) or locally known as “asam jawa” belongs to the family Leguminosae. *T. indica* seeds as by-products from the fruits were previously reported to contain high polyphenolic content. However, identification of their bioactive polyphenols using recent technologies is less well researched but nonetheless important. Hence, it was the aim of this study to provide further information on the polyphenolic content and antioxidant activities as well as to identify and quantify its bioactive polyphenols.

**Methods:**

*T. indica* seeds were extracted with methanol and were then fractionated with different compositions of hexane, ethyl acetate and methanol. Polyphenolic contents were measured using Folin-Ciocalteu assay while antioxidant activities were measured using DPPH radical scavenging and ferric reducing (FRAP) activities. The cytotoxic activities of the crude extract and the active fraction were evaluated in HepG2 cells using MTT assay. The cells were then pre-treated with the IC_20_ concentrations and induced with H_2_O_2_ before measuring their cellular antioxidant activities including FRAP, DPPH, lipid peroxidation, ROS generation and antioxidant enzymes, SOD, GPx and CAT. Analyses of polyphenols in the crude extract and its active fraction were done using UHPLC and NMR.

**Results:**

Amongst the 7 isolated fractions, fraction F3 showed the highest polyphenolic content and antioxidant activities. When HepG2 cells were treated with fraction F3 or the crude extract, the former demonstrated higher antioxidant activities. F3 also showed stronger inhibition of lipid peroxidation and ROS generation, and enhanced activities of SOD, GPx and CAT of HepG2 cells following H_2_O_2_-induced oxidative damage. UHPLC analyses revealed the presence of catechin, procyanidin B2, caffeic acid, ferulic acid, chloramphenicol, myricetin, morin, quercetin, apigenin and kaempferol, in the crude seed extract of *T. indica*. UHPLC and NMR analyses identified the presence of caffeic acid in fraction F3. Our studies were the first to report caffeic acid as the active polyphenol isolated from *T. indica* seeds which likely contributed to the potent antioxidant defense system of HepG2 cells.

**Conclusion:**

Results from this study indicate that caffeic acid together with other polyphenols in *T. indica* seeds can enhance the antioxidant activities of treated HepG2 cells which can provide protection against oxidative damage.

## Background

Oxidative damage has been implicated in ageing and many human diseases and disorders including cancer, cardiovascular diseases especially atherosclerosis, diabetes and chronic inflammatory diseases [1]. Antioxidants can delay or inhibit oxidative damage by acting as free radical scavengers, working primarily by donating electrons to the free radicals, thereby deactivating them [2]. Amongst the sources of antioxidants, natural antioxidants from plants have been gaining attention in recent years due to their therapeutic values and fewer biological side effects. Amongst the numerous phytochemicals, polyphenolic compounds have gained popularity due to their reported potent antioxidant activities [3, 4].

*Tamarindus indica L.* (*T. indica*) or locally known as “asam jawa” is a medicinal plant with high antioxidant activities. The plant belongs to the family Leguminosae and grows indigenously in Africa [5], although it is now cultivated in many tropical countries [6]. The fruit, which is rich in vitamin C and contains tartaric, malic, and citric acids as well as sugars, is the most consumed part of the plant. Due to its sweet-sour taste, the fruit is used as flavoring in cooking. The fruit is used as ingredients in commercialized food products such as drinks, curries, chutneys and also in Worcestershire sauce [7]. On the other hand, tamarind seed is a by-product of the commercial utilization of the fruit. The seeds are also edible and are normally roasted and consumed as pulses [8]. Commercially, tamarind seed is used as raw material for production of tamarind seed gum, for use in industries such as food [9] and medicine, as thickening and stabilizing agent [10, 11]. Scientifically, the aqueous seed extracts of *T. indica* showed potent anti-diabetic and anti-hyperlipidemic activities in streptozotocin (STZ)-induced male rats [12].

Our group had initially reported that the methanol extracts of *T. indica* seed, leaf, leaf veins and skin contained considerable polyphenolic content and antioxidant activities [13]. Amongst these extracts, the methanol seed extract of *T. indica* showed higher polyphenolic content and more potent antioxidant activities. However, this study was performed in the crude extracts which contained mixtures of the polyphenolic antioxidants. As such, the bioactive compounds, particularly the polyphenols, responsible for the antioxidant activities have not been thoroughly researched. In view of this limited data, it was the aim of this study to perform bioactivity-guided fractionation of the crude methanol seed extract of *T. indica* and to identify the bioactive compounds using chromatographic and spectroscopic techniques.

## Methods

### Chemicals

Solvents used for extraction of plant samples were purchased from Fisher Scientific. The polyphenolic standards were of HPLC grade (purity >99 %) and were obtained from Sigma. Water used was of Millipore quality. The human liver HepG2 cell lines were purchased from American Type Culture Collection (ATCC), USA. The growth medium, serum and antibiotics for the cell culture experiments were purchased from Flowlab, Australia. All other reagents used in the experiments were of analytical grade and obtained mostly from Fluka and Sigma.

### Extraction and fractionation of the crude methanol seed extract of *T. indica*

*T. indica* plants were collected from Kedah in the northern region of Malaysia. The voucher specimen of the plant (ID no.: KLU 45976) was deposited in the Rimba Ilmu Herbarium, University of Malaya. The dried and powdered seeds of *T. indica* were extracted with methanol at room temperature for 24 h with a mass to volume ratio of 1:20 (g/ml) [13]. The methanol extract was evaporated under reduced pressure and the residues were dissolved in 10 % DMSO. The two solvents, methanol and DMSO were non-toxic at concentrations that were used to dissolve the plant extracts. Extracts were kept at −20 °C until further analyses.

The methanol seed extract of *T. indica* was subjected to silica gel column chromatography (0.063–0.200 mm) (Merck) and eluted with hexane:ethyl acetate (90:10), (70:30), (50:50), (25:75), (0:100) and ethyl acetate:methanol (25:75), (50:50), (75:25), (0:100), successively. Thirty fractions were subsequently combined into seven sub-fractions according to TLC behaviour when developed using various proportions of ethyl acetate and methanol as the mobile phase. The spots on the TLC sheet were visualized under UV lamp (CAMAG UV, NC, USA) in short wavelength (254 nm) and long wavelength (360 nm).

### Analyses for polyphenolic content

Polyphenolic content of the fractions was assessed using the Folin-Ciocalteu assay, employing gallic acid as the standard [14]. Folin–Ciocalteu reagent (1:10) was added to the plant extracts or standard, mixed and incubated for 5 min at room temperature before the addition of 0.115 mg/ml of Na_2_CO_3_. The mixture was incubated for 2 h before absorbance readings were taken at 765 nm. Results obtained were expressed as mg gallic acid equivalents (GAE)/g dried plant material.

### Ferric reducing activity

The ferric reducing activity of the plant extracts was estimated based on the FRAP assay developed by Benzie and Strain (1996) [15].

Reagents for this assay consisted of 300 mmol/l acetate buffer (pH 3.6), 10 mmol/l 2,4,6-tri(2-pyridyl)-S-triazine (TPTZ) in 40 mmol/l of HCl and 20 mmol/l FeCl_3_.6H_2_O. FRAP reagent was prepared fresh by mixing the respective reagents in a ratio of 10:1:1. The assay was performed by initially incubating the FRAP reagent at 37 °C for 5 min and subsequently taking a blank reading at 593 nm. Thereafter, 30 μl of plant extracts or standard along with 90 μl of distilled water was added to 900 μl of FRAP reagent. Absorbance readings were recorded immediately upon addition of the FRAP reagent and after 4 min of reaction. FeSO_4_ was used as the standard and was tested in parallel.

### DPPH radical scavenging activity

The scavenging activity of the free radical, 2,2-diphenyl-l-picrylhydrazyl (DPPH) was determined by the method described by Braca *et al*. (2001) [16]. A series of concentrations of the plant extracts were added to DPPH solution (0.04 mg/ml). After a 20 min incubation period, absorbance was read at 517 nm. Radical scavenging activity against DPPH was expressed as percentage of inhibition and this was calculated according to the following formula:

$$ \mathrm{Percentage}\ \mathrm{of}\ \mathrm{inhibition}\ \left(\%\right) = \frac{\left[\mathrm{O}\mathrm{D}\ \mathrm{control}\ \hbox{-}\ \mathrm{O}\mathrm{D}\ \mathrm{sample}\right]}{\mathrm{OD}\ \mathrm{control}}\times 100\%,\ \mathrm{where}\ \mathrm{O}\mathrm{D} = \mathrm{O}\mathrm{ptical}\ \mathrm{D}\mathrm{ensity} $$

Using the percentage of inhibition values, a dose-response curve was plotted, from which the IC_50_ value was extrapolated. The antioxidant activity was expressed as IC_50_ value which was the concentration (μg/ml) that inhibited the DPPH radicals by 50 %. From the polyphenolic content and antioxidant analyses, the most active fraction was identified and was subjected to cell culture treatments.

### Analyses of antioxidant activities in HepG2 cells treated with the antioxidant-rich crude and fractionated seed extracts of *T. indica*

#### Cytotoxicity assay

The human liver HepG2 cells were grown in Dulbecco’s modified Eagle’s medium (DMEM) supplemented with 10 % fetal bovine serum, 1 % penicillin and 1 % streptomycin. Cells were maintained in humidified air with 5 % CO_2_ at 37 °C.

A cytotoxicity assay was then carried out using 3-(4,5-dimethylthiazol-2-yl)-2,5-diphenyltetrazolium bromide (MTT) as described by Mosmann (1983), with minor modifications. HepG2 at a density of 5000 cells per well were seeded in a 96-well ELISA microplate and incubated at 37 °C in 5 % CO_2_ for 24 h. After 24 h, plant extracts, at various concentrations ranging from 0 to 200 μg/ml, were added into the wells. The cells were left to grow in the incubator for 48 h. After 48 h, MTT reagent was added and the mixture was further incubated for 4 h. Then, the mixture in each well was removed and formazan crystals formed were dissolved in 75 % isopropanol. Spectrophotometry measurement of the mixture was performed using a microplate-reader (Bio-Rad) at wavelengths of 570 and 620 nm. A log plot of cell viability (%) against the concentrations of plant extracts was constructed with the final concentration ranging from 0 to 200 μg/ml. From the plot, the final concentration of the plant extracts that reduced cell viability by 50 % (IC_50_) was calculated. The relatively non-toxic IC_20_ concentration where the HepG2 cells showed more than 80 % viability was also calculated. This concentration (IC_20_) was selected for further treatment in HepG2 cells.

### Treatment of HepG2 cells with the crude methanol and fractionated seed extracts of *T. indica*

Confluent (85–90 %) HepG2 cells (1 × 10^6^ cells), which were maintained in DMEM, were treated with the antioxidant-rich crude and fractionated extracts of *T. indica* seeds at IC_20_ concentration, determined from the MTT assay. The cells were incubated with or without the extracts at 37 °C for 24 h. After 24 h incubation, the cell lysates were washed with cold PBS and lysed in Tris-Hydrochloride (Tris-HCl) buffer (25 mM, pH 7.4) and subsequently sonicated for 2 min at 70 % amplitude. The supernatant was used for analyses of antioxidant activities, antioxidant enzymes, lipid peroxidation and ROS levels.

### FRAP activity in treated HepG2 cells

The protocol for FRAP assay measured in HepG2 cells was as described above, with slight modifications [17]. Briefly, 87.5 μl of freshly prepared FRAP reagent was added to the cells and the reaction mixture was incubated for 30 min at 37 °C. Absorbance reading was then taken at 590 nm. FeSO_4_.7H_2_O was used as standard and was run parallel with the samples.

### DPPH radical scavenging activity in treated HepG2 cells

The DPPH radical scavenging activity was determined following the method described above. Freshly prepared DPPH reagent with a volume of 175 μl was added to the cells and absorbance was read at 517 nm after 20 min. Results were expressed as IC_50_ of the plant extracts determined from the dose-response curve.

### Inhibition of lipid peroxidation and ROS production and activity of antioxidant enzymes in treated HepG2 cells

HepG2 cells were seeded and pre-treated with the extracts and fractions following the previously described protocol. After 24 h, oxidative stress was induced in the cells with 1 mM H_2_O_2_ for 2 h. Untreated cells induced with H_2_O_2_ acted as control. The cells were subsequently washed with ice-cold PBS and detached using a scraper. Cells were then collected into a microtube and centrifuged for 10 min at 8000 × g at 4 °C. The supernatant was discarded and the cell pellets were lysed in Tris-HCl buffer (25 mM, pH 7.4) followed by sonication for 5 min at 60 % amplitude. Protein content of each sample was quantified using Bradford assay, employing bovine serum albumin (BSA) as the standard. One hundred μg/ml of protein was used in the lipid peroxidation and antioxidant enzyme assays.

### Bradford protein assay

Protein concentration was estimated using Bradford Protein Assay Kit I (Bio-Rad, USA) with Bovine Serum Albumin (BSA) as the standard. Briefly, the BSA stock (2 mg/ml) was diluted to 1 mg/ml. Two hundred microlitres of Bradford dye reagent was pipetted into each well of a 96-well dish. The diluted BSA stock was added to the wells to give a final concentration within the range of 0.2l–1 mg/ml. One microlitre of sample was then added to the wells and absorbance was read at 595 nm after 5 min incubation at room temperature. A BSA standard curve was generated for estimation of protein concentration of the samples. Samples corresponding to 100 μg/ml of protein were subsequently aliquoted into microcentrifuge tubes for the lipid peroxidation and antioxidant enzyme assays.

### Inhibition of lipid peroxidation

Levels of lipid peroxidation in the treated cells were determined by measuring the production of malondialdehyde (MDA) in the presence of thiobarbituric acid (TBA) [18]. The TBA reagent used in this assay consisted of a mixture of TBA, trichloroacetic acid (TCA) and 70 % perchloric acid (HClO_4_) in distilled water. Tetraethoxypropane (TEP) in ethanol was used as the standard. TBA reagent was added to the sample or standard and boiled for 20 min. The mixture was left to cool and was centrifuged for 10 min at 960 × g at 25 °C. The supernatant was pipetted into a 96-well plate and absorbance was measured at 532 nm on a microplate reader. The amount of MDA in the treated and untreated samples was determined using the equation obtained from the standard curve of TEP. Results were expressed as nmol MDA equivalents/mg of protein.

The abundance of 4-hydroxynonenal (4-HNE) protein adducts in both untreated and treated cells were also measured using ELISA kit to analyse the effect of the crude extract and fraction F3 of *T. indica* seeds in inhibiting lipid peroxidation in HepG2 cells. Supernatants were collected from each sample and the abundance of proteins were detected against 4-HNE (QY-E05206) according to the manufacturers’ protocols (Shanghai Qayee, China). Briefly, 10 μl of samples were pipetted into a pre-coated ELISA microplate, followed by the addition of HRP conjugate for detection. The plate was washed to remove unbound substances and the end-product was measured at 450 nm. A known HNE-BSA standard curve was constructed to determine the concentration of 4-HNE protein adducts present in the samples.

### Assay for reactive oxygen species (ROS)

ROS production was assessed using the method of Wang and Joseph (1999) with minor modifications [19]. HepG2 cells were plated into black 96-well plates, seeded and treated as previously described. At the end of the treatment, the medium was removed and the cells were washed with PBS and then incubated with 100 mM dichlorofluorescein diacetate (DCF-DA) in PBS for 30 min at 37 °C. The formation of the fluorescent-oxidized derivatives of DCF-DA was monitored at emission wavelength of 530 nm and excitation wavelength of 485 nm in a fluorescence multi-detection reader (Synergy HTTM Multi-detection microplate reader; BioTek Instruments Inc, VT, USA). ROS production was expressed as relative fluorescence unit (RFU) produced by DCF-DA/mg of total protein [20].

### Antioxidant enzyme assays

The antioxidant enzymes tested were superoxide dismutase (SOD), catalase (CAT) and glutathione peroxidase (GPx). All three enzyme activities were measured using assay kits from Cayman Chemicals (USA), following the manufacturer’s instructions.

SOD assay utilizes tetrazolium salt for detection of superoxide radicals generated by xanthine oxidase and hypoxanthine. The SOD activities were estimated colorimetrically at a wavelength of 450 nm. SOD activities were expressed as Unit/mL, following the manufacturer’s protocol, whereby one unit is defined as the amount of enzyme needed to exhibit 50 % dismutation of the superoxide radicals per ml of reaction volume (Unit/mL).

CAT assay measures the reaction of CAT in the presence of an optimal concentration of H_2_O_2_. The reaction produced formaldehyde, which was measured colorimetrically with 4-amino-3-hydrazino-5-mercapto-1,2,4-triazole (Purpald) as the chromogen. Purpald specifically forms a bicyclic heterocycle with aldehydes, which upon oxidation changes from colorless to purple. The formaldehyde concentration in the cells was measured using the equation obtained from the linear regression of the standard curve. CAT activities in the samples were expressed in nmol H_2_O_2_ oxidized/min/ml.

GPx activity was measured through a coupled reaction with glutathione reductase whereby GPx catalyzed the reduction of hydroperoxides, including H_2_O_2_ by reduced glutathione, which led to the protection of cells from oxidative damage. GPx activities in the cells were initially calculated by determining the changes in absorbance per minute obtained from the standard curve of GPx control. One unit of activity is defined as the amount of enzyme that caused the oxidation of 1.0 nmol of NADPH to NADP^+^ per minute per ml of sample (nmol/min/ml) at room temperature.

### Analysis of polyphenols using ultra high performance liquid chromatography (UHPLC)

#### Preparation of samples

The crude and fractionated seed extracts of *T. indica* were subjected to acid hydrolysis prior to analysis by UHPLC to release glycosides from the conjugated polyphenols [21]. The dried powder of *T. indica* leaf and its fraction were mixed with 1.2 M HCl in 50 % aqueous methanol containing 20 mM sodium diethyldithiocarbamate as an antioxidant. The mixture was heated at 90 °C for 2 h using a heating block with stirring capacity (Reacti-Therm, Pierce, Rockford, USA). Aliquots of the samples were taken after the hydrolysis, centrifuged for 5 min at 5000 × g and diluted with distilled water (pH 2.6). The hydrolysate was then filtered through a 0.20 μm polytetrafluoroethylene (PTFE) membrane filter prior to analysis by UHPLC.

Analyses of polyphenols were conducted using an UHPLC 1290 Infinity LC system (Agilent Technologies, Waldbronn, Germany) equipped with a binary pump, diode array detector and an auto sampler. Separation of polyphenols was achieved using a ZORBAX Eclipse Plus C_18_ column (50 × 2.1 mm, 1.8 μm) (Agilent, Germany), with temperature of the auto sampler set at 10 °C. Three microlitres of the sample were injected into the system. The mobile phase consisted of water containing 0.1 % trifluoroacetic acid (TFA), pH 2.6 (solvent A) and methanol (solvent B). Optimum separation was achieved using a constant flow rate of 0.4 mL/min and maximum back pressure of 4.000 psi, with the following gradient: 95 to 85 % A in 2 min, 85 to 75 % A in 1 min, 75 to 40 % A in 1 min, 40 to 0 % A in 0.2 min, 0 % A in 0.6 min, and 0 to 95 % A in 0.2 min, followed by a re-equilibration time of 2 min. The polyphenols were detected at 254, 280 and 325 nm on a diode array detector. Concentration of the polyphenolic compounds in the sample was estimated based on the standard curve generated from pure polyphenolic standards ran under the same conditions as described above.

### Identification of polyphenolic compounds in fraction F3 using Nuclear Magnetic Resonance (NMR)

Fraction F3 was initially concentrated and vacuum-dried using a SpeedVac (Thermo Scientific, DE, USA). Fifteen milligrams of the powder was dissolved in 0.7 mL of methanol-d_4_ in a 5 mm NMR tube for NMR spectra analyses.

NMR spectra were generated from JNM Lambda-400 MHz FT-NMR and ECA-400 FT-NMR spectrometer (Jeol, USA), at ambient temperature. Methanol-d_4_ was used as a solvent with tetramethylsilane (TMS) as the internal standard. All spectra were reported in units of ppm on the scale, relative to TMS (δ 0.00) and the coupling constants are given in Hz. Structure elucidation of the bioactive compound was based on ^1^H NMR, ^13^C NMR, H-H Correlation Spectroscopy (H-H COSY), Distortionless Enhancement of Polarization Transfer using a 135° decoupler pulse (DEPT-135) and Heteronuclear Single Quantum Coherence (HSQC) using ACD/NMR Processor Academic Edition software (ACD Labs, Toronto, Canada) [22].

### Statistical analyses

All analyses were done in triplicate. Results were expressed as means ± standard deviation. The data were statistically analyzed using SPSS statistical software for Windows, Version 21.0. (Armonk, NY: IBM Corp.) An independent t-test was used for comparisons of means between groups. One-way analysis of variance (ANOVA) and Tukey’s Honestly Significant Difference test were used to compare means among groups. Pearson correlation coefficient was used to determine the relationship between polyphenolic content in the plant extracts with the respective antioxidant activities. The level of significance was set at *p* < 0.05.

## Results

### Yield, polyphenolic content and antioxidant activities of the crude and fractions

Table 1 shows the extraction yield of the fractions isolated from the methanol seed extract of *T. indica*, which ranged from 8 to 210 mg/g dried weight. Amongst all the fractions, fraction 3 (F3) had the highest yield while fraction 6 (F6) had the least. Amongst the seven fractions, fraction F3 contained the highest polyphenolic content (572 ± 3.78 GAE mg/g), followed by F2 (445 ± 3.97 GAE mg/g) (Table 1). Both F3 and F2 had higher polyphenolic content than the crude extract. F3 also showed the most potent ferric reducing and DPPH radical scavenging activities. DPPH radical scavenging activity, expressed as IC_50_ could only be detected in fraction F2 and F3. F3 had even lower IC_50_ value than the crude extract. Fractions 5, 6, and 7 had very low polyphenolic content (<20 mg GAE/G) and antioxidant activities whereby ferric reducing activities were not detected in all three extracts.

**Table 1:** Yield, polyphenolic content and antioxidant activities of the crude methanol seed extract of *T. indica* and the corresponding fractions

Extracts	Yield (mg/g dried weight)	Polyphenolic content (GAE mg/g)	Ferric reducing activity (mmol/g dried weight)	DPPH• radical scavenging activity (IC_50_ in μg/ml)
Crude				
Seeds	240.72	271.23 ± 4.78^a^	1.45 ± 0.018^a^	50.23 ± 0.37^a^
Fraction				
1	20.80	84.25 ± 12.38^b^	0.52 ± 0.003^b^	ND
2	107.28	444.57 ± 3.97^c^	2.30 ± 0.001^c^	60.76 ± 0.45^b^
3	209.52	571.71 ± 3.78^d^	5.50 ± 0.001^d^	42.31 ± 0.32^c^
4	56.60	141.81 ± 4.59^e^	1.08 ± 0.01^e^	ND
5	10.13	15.58 ± 1.03^f^	ND	ND
6	6.76	10.38 ± 0.60^g^	ND	ND
7	8.29	11.49 ± 0.15^h^	ND	ND

^a-h^ Values not sharing the same superscript lowercase letter within the same column are significantly different at *p* < 0.01

Results are average of triplicate analyses ± S.D

*ND* not detected

### Correlation analyses

Pearson correlation analyses were performed to assess the relationship between the polyphenolic content of the isolated fractions and their respective antioxidant activities. A strong positive correlation was observed between polyphenolic content and DPPH radical scavenging activity (R^2^ = 0.951) and with ferric reducing activity (R^2^ = 0.911) (*p* < 0.05). Hence, the polyphenolic content of the fractions contributed significantly to their antioxidant properties.

### Cytotoxicity and antioxidant analyses in HepG2 cells

#### Cytotoxicity

**Fig. 1:**
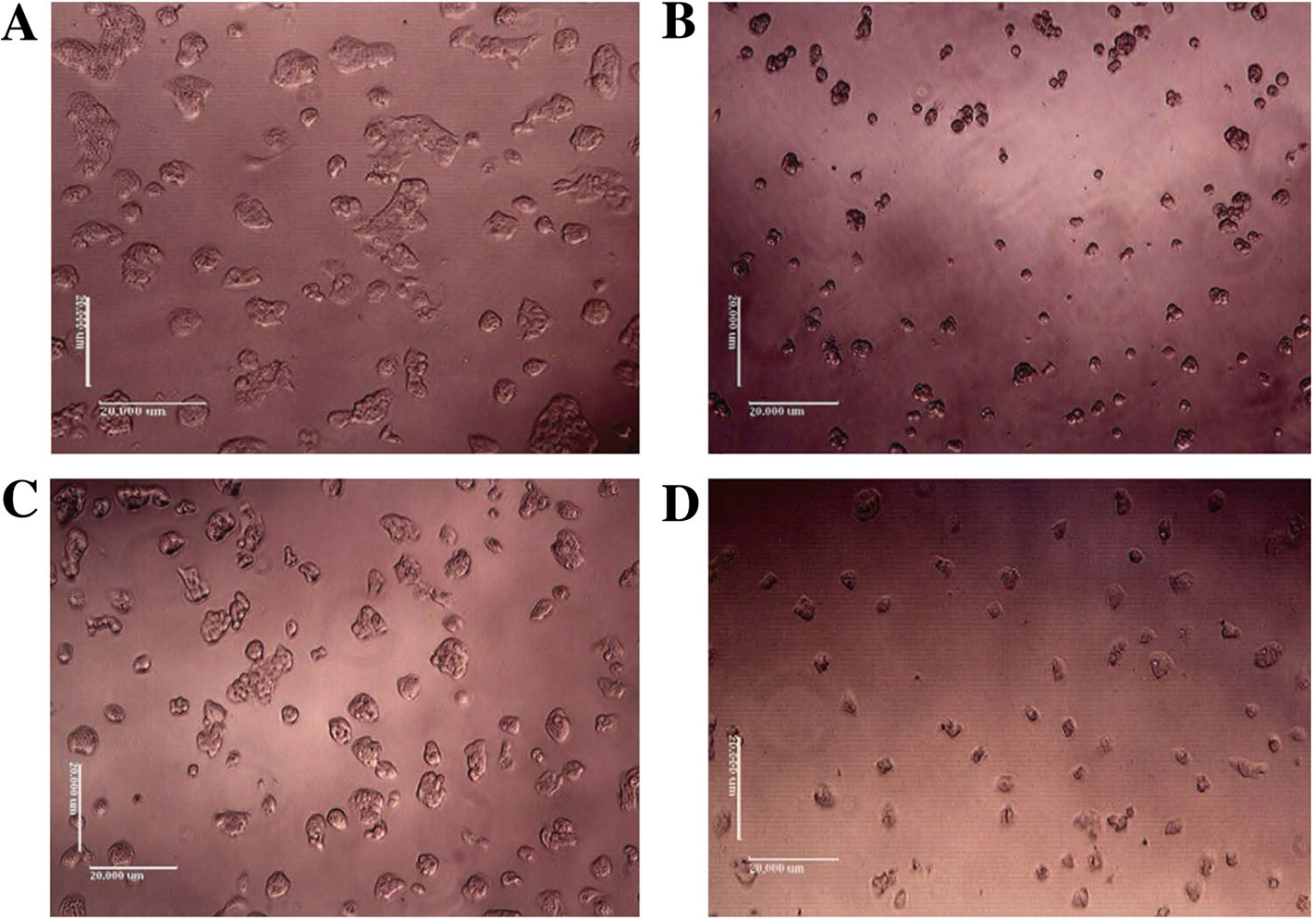
**a-d** Morphological changes of HepG2 cells in response to treatment with IC_20_ and IC_50_ concentrations, respectively, of the crude methanol seed extract (**a-b**), and its active fraction, F3 (**c-d**). The images were captured using a digital camera attached to an inverted microscope

The cytotoxic effects of the crude methanol seed extract and fraction F3 on HepG2 cells were expressed as IC_50_ (Table 2), which signify the concentration of the plant extracts needed to inhibit 50 % of cell viability after 24 h of treatment. Following fractionation of the crude extract, F3 exhibited higher cytotoxic effects on HepG2 cells with an IC_50_ of 50.63 ± 0.21 μg/ml as compared to the crude seed extract (IC_50_ = 104.71 ± 0.07). The non-toxic IC_20_ concentration where the HepG2 cells showed more than 80 % viability was also calculated. This concentration (IC_20_) was selected for further treatment in HepG2 cells. Figure 1a-d shows the images of the cells viewed under light microscope, when the cells were treated with IC_50_ and IC_20_ concentrations of the crude methanol seed extract and fraction F3. Generally, at IC_20_, HepG2 cells adhered to the flasks, were spindle-shaped with defined cell membranes. However, at IC_50_, cells were shrunken, clumped together and floated in the medium. The relatively non-toxic IC_20_ concentration of the extracts of which the HepG2 cells showed more than 80 % viability was then selected for further analyses.

**Table 2:** Cytotoxic effects and antioxidant activities of the crude methanol seed extract and fraction F3 of *T. indica* in HepG2 cells

Sample	IC_50_ value (μg/ml)	IC_20_ value (μg/ml)	FRAP (mmol/g dried weight)	DPPH (IC_50_ in μg/ml)
Seed-treated	104.71 ± 0.07^a^	35.43 ± 0.08^a^	14.41 ± 0.23^a^	40.23 ± 3.53^a^
F3-treated	50.63 ± 0.21^b^	12.12 ± 0.03^b^	18.09 ± 0.54^b^	20.89 ± 2.76^b^

^a,b^ Values not sharing the same superscript lowercase letter within the same column were significantly different at *p* < 0.01

Results are expressed as average of triplicate analyses ± S.D

### Cellular FRAP and DPPH radical scavenging

The effects of the crude methanol extracts of seeds and fraction F3 on cellular antioxidant activities in HepG2 cells were determined following treatment of the cells with the IC_20_ concentration of the extracts.

Untreated HepG2 cells showed very low ferric reducing activity whereas the DPPH radical scavenging activity was undetected. Fractionation of the crude seed extract resulted in higher antioxidant activities (Table 2). F3 showed more potent ferric reducing and DPPH radical scavenging activities in the treated HepG2 cells compared to its crude extracts.

### Lipid peroxidation

**Fig. 2:**
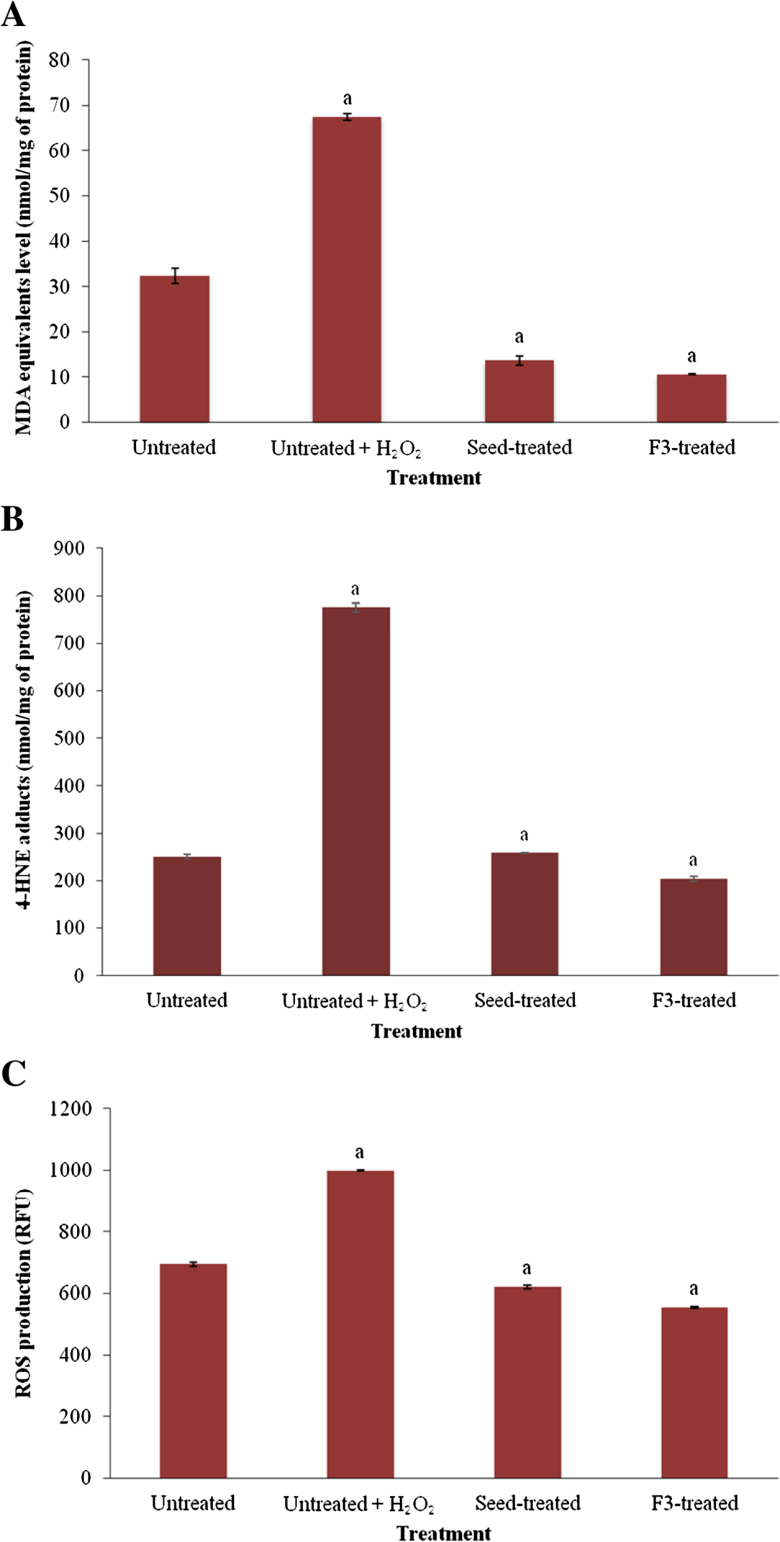
**a-c** The effects of the crude methanol seed extract and fraction F3 from *T. indica*, on lipid peroxidation, measured as MDA levels (**a**) and 4-HNE adducts abundance (**b**), and inhibition of ROS production (**c**) in HepG2 cells. Results for MDA levels were expressed as nmol MDA equivalents/mg of protein while 4-HNE levels were expressed as nmol 4-HNE adducts/mg protein. ROS production was measured using fluorescence multi-detection microplate reader and the results were expressed as Relative Fluorescence Unit (RFU). ^a^ indicates significant difference from untreated cells (*p* < 0.05). MDA- malondialdehyde; 4-HNE - 4-hydroxynonenal; ROS- reactive oxygen species

H_2_O_2_-induced oxidative damage in the untreated HepG2 cells caused approximately 2-fold increase in MDA equivalents levels compared to untreated and uninduced cells (Fig. 2a). However, pre-treatment of HepG2 cells with the crude seed extract and F3 caused significant inhibition of lipid peroxidation (*p* < 0.05), with MDA levels being lower than the untreated and uninduced cells. F3 and its crude seed extract reduced MDA levels by 84 and 79 %, respectively, compared to the untreated cells induced with H_2_O_2_.

Figure 2b shows the detection of altered abundance of 4-HNE adducts in HepG2 cells treated with the crude seed extract and fraction F3 of *T. indica* using ELISA analyses. H_2_O_2_-induced HepG2 cells showed approximately 3-fold increase in levels of 4-HNE compared to untreated and uninduced cells. Pre-treatment of the cells with the crude seed extract led to reduction of 4-HNE levels similar to the untreated, uninduced cells or in the case of fraction F3, even lower than the untreated cells.

#### ROS production

**Fig. 3:**
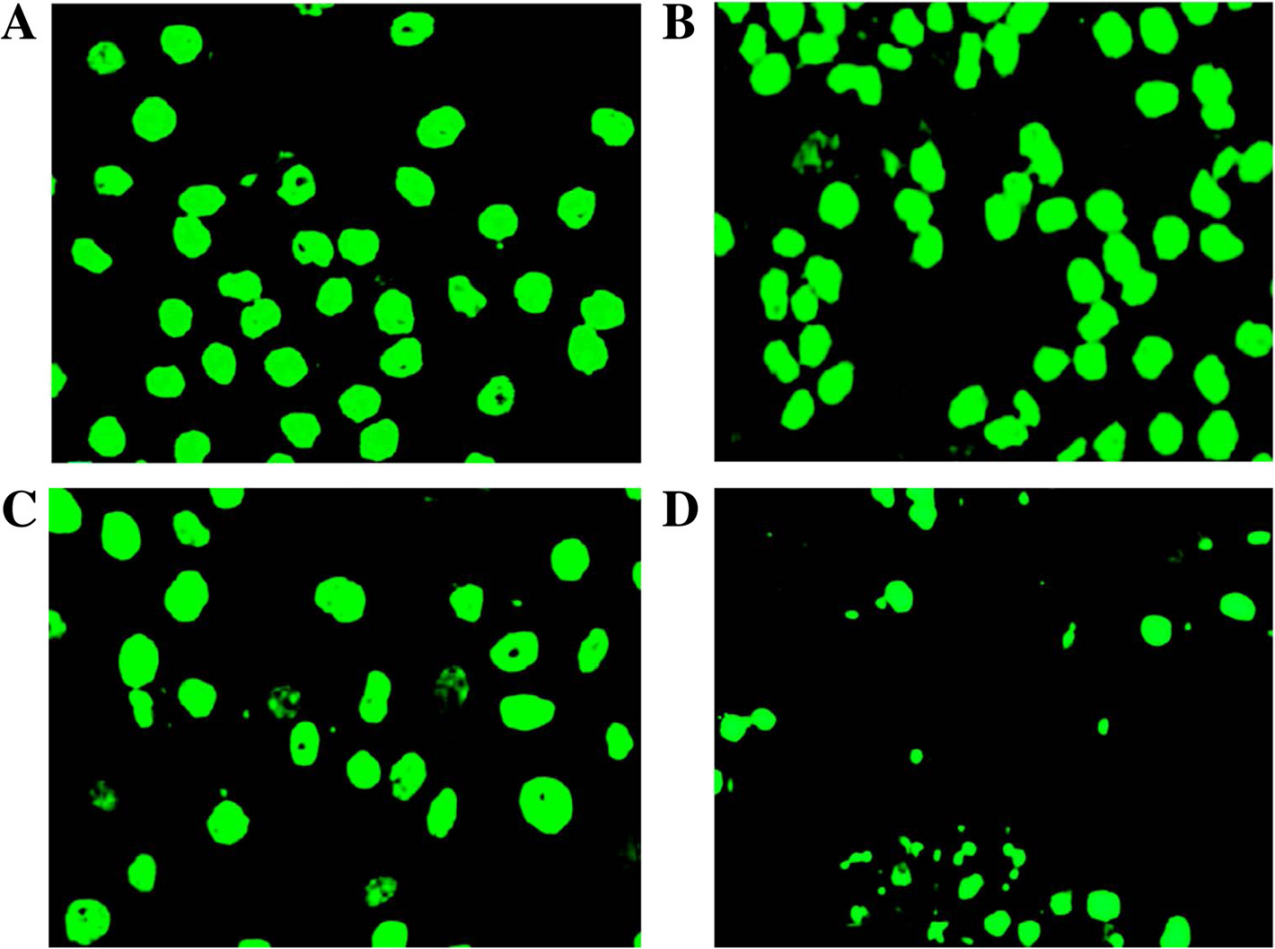
**a-d** Image-based ROS measurement captured by fluorescence microscopy in HepG2 cells; **a** Untreated and uninduced cells, **b** Untreated and H_2_O_2_-induced cells, **c** Seed-pre-treated and H_2_O_2_-induced cells and **d** F3-pre-treated and H_2_O_2_-induced cells

The effect of the methanol seed extract and fraction F3 on ROS production was evaluated in HepG2 cells using a cell permeable probe, DCF-DA (Fig. 2c). The fluorescence intensity is proportional to the amount of peroxides, which are produced by the cells. ROS production increased significantly after HepG2 cells were challenged with 1 mM H_2_O_2_ as compared with the untreated and unchallenged cells. However, pre-treatment of the cells with IC_20_ concentration of the seed extract and fraction F3 significantly decreased H_2_O_2_-mediated ROS formation, whereby intensity of the fluorescence was reduced to a level lower to that of the untreated, H_2_O_2_-challenged cells. Additionally, F3 showed higher inhibition of ROS compared to the crude seed extract (p < 0.05). This can also be observed when ROS production was captured by fluorescence microscopy (Fig. 3a-d).

### Antioxidant enzymes

**Fig. 4:**
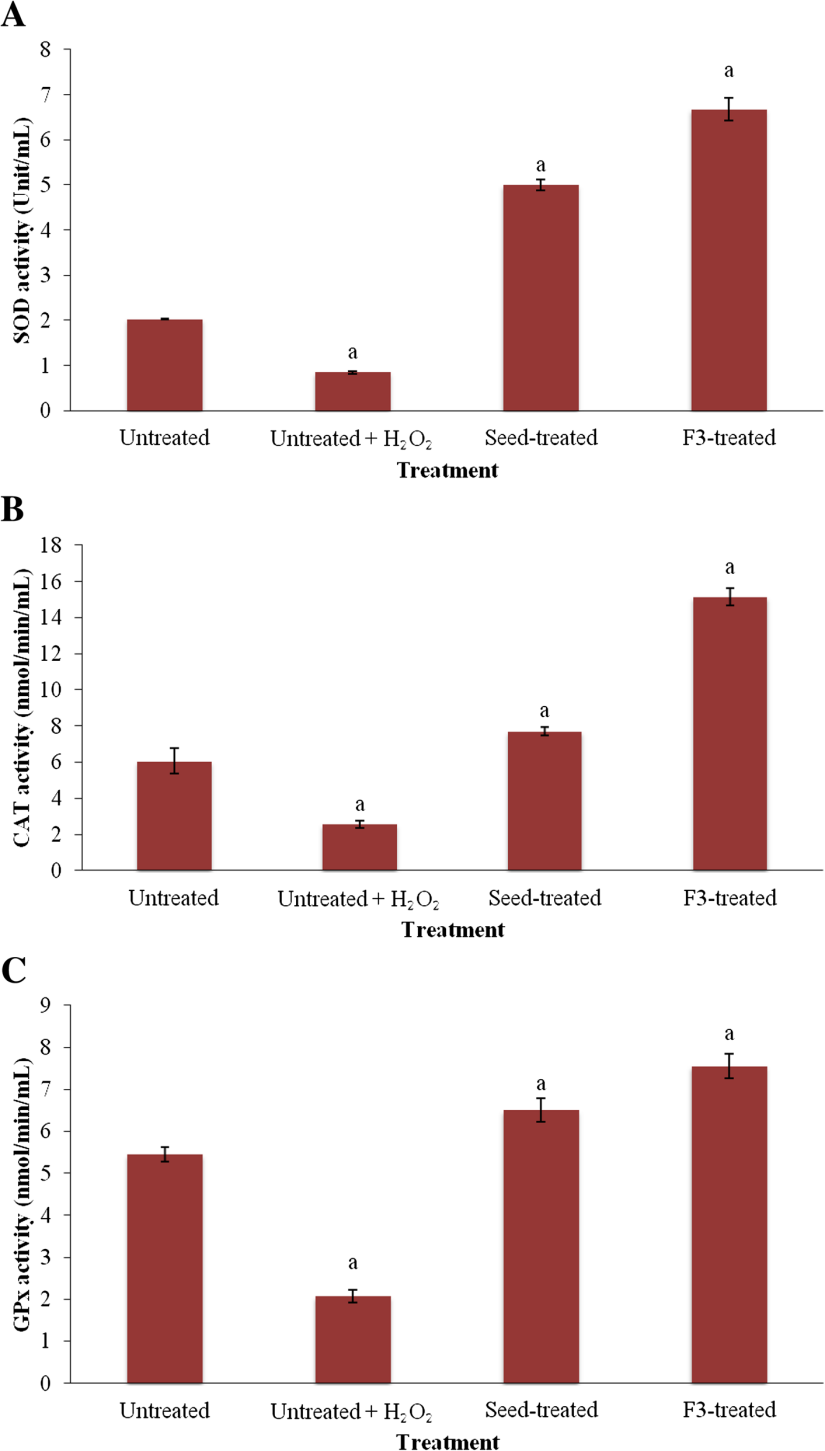
**a-c** The effects of the crude methanol seed extract and fraction F3 from *T. indica* on superoxide dismutase (**a**), catalase (**b**) and glutathione peroxidase (**c**) activities. ^a^ indicates significant difference from untreated cells (*p* < 0.05). SOD- superoxide dismutase, CAT- catalase, GPx-glutathione peroxidase

As shown in Fig. 4a-c, H_2_O_2_-induced oxidative damage in the untreated HepG2 cells caused reduction of all three antioxidant enzymes compared to control untreated cells. However, pre-treatment of HepG2 cells with fraction F3 and the crude extract prior to induction of oxidative damage significantly increased the activities of SOD, GPx and CAT. The increase was higher compared to the untreated cells as well as the untreated + H_2_O_2_ cells. Additionally, F3 showed higher antioxidant enzyme activities compared to the crude seed extract (*p* < 0.05).

### Analyses of polyphenols in the methanol seed extract of *T. indica* and fraction F3 using UHPLC

**Fig. 5:**
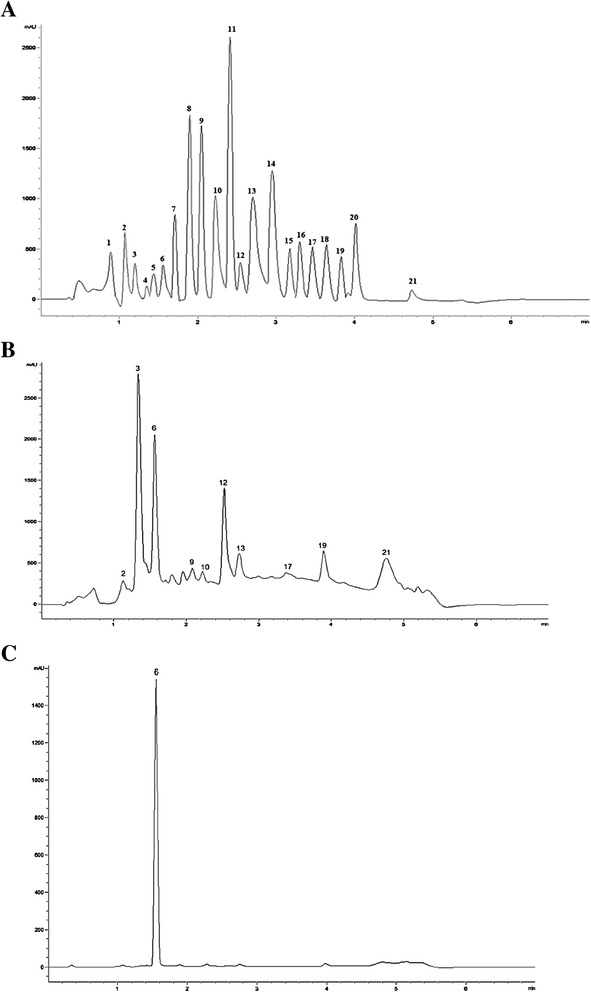
**a-c** Chromatograms of polyphenolic standards (**a**), methanol seed extract of *T. indica* (**b**) and fraction F3 (**c**). Individual retention times and peak identification of the polyphenols in the extracts were compared with the chromatogram of a standard mixture of polyphenols analyzed with UHPLC 1290 Infinity LC system. The polyphenols were detected at a wavelength of 280 nm. 1: gallic acid; 2: catechin; 3: procyanidin B2; 4: chlorogenic acid; 5: epicatechin; 6: caffeic acid; 7: vanillic acid; 8: p-coumaric acid; 9: ferulic acid; 10: chloramphenicol; 11: rutin; 12: myricetin; 13: morin; 14: ellagic acid; 15: cinnamic acid; 16: naringenin; 17: quercetin; 18: luteolin; 19: apigenin; 20: isorhamnetin; 21: kaempferol

Figure 5a shows the chromatogram of a standard mixture of polyphenols analyzed with the UHPLC system. The polyphenols were detected at a wavelength of 280 nm. A satisfactory separation of the polyphenols was obtained using the previously described gradient system and mobile phases.

Figure 5b shows the chromatogram of the polyphenolic profiles in the crude methanol seed extract of *T. indica*. The chromatogram shows the presence of catechin, procyanidin B2, caffeic acid, ferulic acid, chloramphenicol, myricetin, morin, quercetin, apigenin and kaempferol, detected at 280 nm. When fraction F3 was analyzed on the UHPLC, a single peak was observed and this was later identified as caffeic acid based on comparison with the retention time of caffeic acid standard as well as its absorption spectra on the diode array detector (Fig. 5c).

Quantification of the polyphenols detected in the methanol seed extract of *T. indica* and F3 is shown in Table 3. The highest amount of polyphenols in the crude extract was procyanidin B2 followed by caffeic acid and myricetin. The crude extract also contained considerable amounts of apigenin, kaempferol and morin, within the range of 70-115 μg/g. On the other hand, quercetin showed the lowest content in the crude extract. The content of caffeic acid in fraction F3 is almost similar to that in the crude extract.

**Table 3:** Identification and quantification of polyphenols in the crude methanol seed extract and fraction F3 of *T. indica*

Peak number	Compounds	R^2^	Recovery (%)	Polyphenol content (μg/g crude extract)
Crude extract				
2	Catechin	0.9993	101.03	46.92 ± 1.86
3	Procyanidin B2	0.9963	101.44	516.95 ± 4.64
6	Caffeic acid	0.9949	100.24	446.58 ± 2.35
9	Ferulic acid	0.9933	101.45	35.82 ± 1.28
10	Chloramphenicol	0.9980	101.98	27.10 ± 1.33
12	Myricetin	0.9921	101.52	298.69 ± 2.07
13	Morin	0.9911	101.77	70.98 ± 1.35
17	Quercetin	0.9968	98.06	11.48 ± 0.69
19	Apigenin	0.9997	100.25	115.00 ± 2.69
21	Kaempferol	0.9977	98.36	111.41 ± 1.72
Fraction F3				
6	Caffeic acid	0.9949	100.24	451.03 ± 2.84

### Structure elucidation of polyphenol in fraction F3 using NMR

**Fig. 6:**
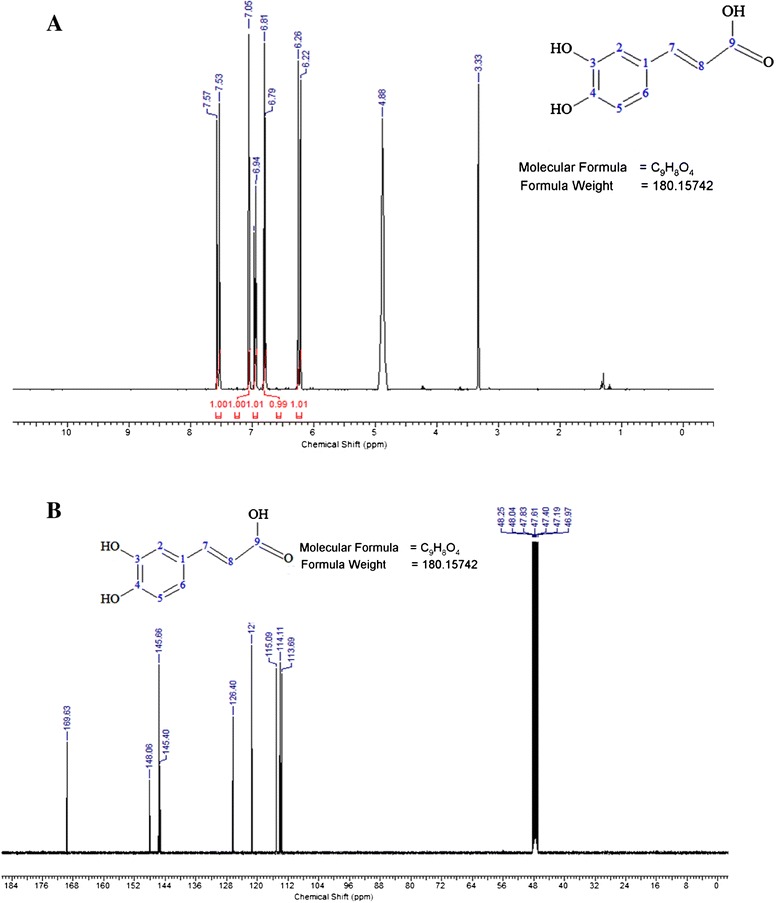
**a** ^1^H NMR and **b** ^13^C NMR spectrum of caffeic acid isolated from fraction F3 from the crude methanol seed extract of *T. indica*

The compound in fraction F3 appeared as a solid, yellow amorphous. NMR analyses identified caffeic acid as the bioactive compound in fraction F3 further confirming the earlier identification by UHPLC. The structure of bioactive compound in F3 was elucidated using ACD/NMR software based on ^1^H, ^13^C, H-H COSY, DEPT-135 and HSQC (ACD Labs, Toronto, Canada) (Fig. 6a and b).

## Discussion

The present study describes the fractionation of antioxidant-rich methanol seed extract of *T. indica* and the subsequent identification of polyphenols in the crude extracts as well as in fraction F3. We had previously shown that amongst the different aerial parts of the *T. indica* plant, the seeds contained considerable amounts of polyphenolic compounds and antioxidant activities, hence the seeds were chosen for fractionation [13]. Polyphenols are secondary plant metabolites, which are proven to have various biological properties including anti-bacterial, anti-inflammatory and anti-allergic activities [23, 24]. Many of these reported activities can be attributed to the antioxidative properties of the polyphenols [25–27].

Of the seven fractions obtained, only the first four fractions showed significant levels of antioxidant activities and correlation analyses confirmed that the polyphenolic content contributed to the antioxidant activities of the respective fractions. Various studies have reported positive correlations between polyphenolic content and antioxidant activities of plants [28, 29] and results from this study further strengthen the impact of polyphenols as potent antioxidants.

In the treatment of the HepG2 cells, the IC_20_ concentration of the plant extract and fraction was used in order to keep the concentration low while still maintaining cell viability above 80 %. As dietary antioxidants are rapidly metabolized and are usually present in small amounts in the circulation, hence using low concentration would be more significant [30]. Our group had previously reported that the IC_20_ concentration of the methanol fruit pulp extract of *T. indica* was able to regulate a sizable number of genes involved in different pathways in liver HepG2 cells, further showing that low concentration of the *T. indica* extract was adequate to induce changes in the cells [31].

Treatments of HepG2 cells with the crude extract and fraction F3 increased antioxidant activities of the cells, increased antioxidant enzymes and inhibited ROS production thus inhibited lipid peroxidation. In all instances, fraction F3 showed higher activities than the crude extract. We hypothesize that the crude extract and fraction F3 were able to protect HepG2 cells against oxidative damage via several mechanisms. The increased FRAP and DPPH radical scavenging activities indicated their abilities to act as reducing agents and radical scavengers, thus removing ROS and preventing oxidative damage. Furthermore, the increased antioxidant enzyme activities indicated increased capacity of the cells to convert free radicals into non-reactive forms. This was reflected in the reduced levels of lipid peroxidation.

Lipid peroxidation is an important factor in the pathologies of many diseases associated with oxidative stress [32]. The reaction of ROS such as H_2_O_2_ with the double bonds of polyunsaturated fatty acids during oxidative damage yield lipid hydroperoxides, which can cause membrane dysfunction. Lipid hydroperoxides can be further metabolized to produce a variety of aldehydes, including malondialdehyde (MDA) and 4-hydroxynonenal (4-HNE) [33, 34]. MDA appears to be the most mutagenic product of lipid peroxidation, whereas 4-HNE is the most toxic one [35]. Both MDA and 4-HNE are biomarkers for oxidative stress and are found to be elevated in various diseases thought to be related to oxidative damage [36]. Hence they are widely used as indexes of lipid peroxidation [37]. It has been reported that male broilers fed with polyphenols extracted from *T. indica* seed coat had lower MDA levels [38]. In addition, *T. indica* seed coat was also reported to attenuate lipid peroxidation in H_2_O_2_-induced human foreskin fibroblast cells [39]. Treatment with the crude extract and particularly fraction F3 caused significant inhibition of lipid peroxidation in oxidative damage-induced HepG2 cells. Hence, it can be speculated that the crude seed extract and fraction F3 mediated protection of HepG2 cells against toxicity towards H_2_O_2_ by the inhibition of hepatic lipid peroxidation, supporting previous reports.

SOD, GPx and CAT are the primary antioxidant enzymes in mammalian cells that are important in oxygen metabolizing cells. SOD catalyzes the dismutation of superoxide anion radicals into H_2_O_2_ and water, while CAT and GPx convert H_2_O_2_ into water, reducing the amounts of H_2_O_2_, hence protecting the cells against oxidative damage. The increased antioxidant enzyme activities, together with increased FRAP and DPPH radical scavenging activities, could have caused lower production of ROS, leading to inhibition of lipid peroxidation, which was reflected in the lower MDA and HNE amounts.

Changes in the activity of antioxidant enzymes can be considered as biomarkers of the antioxidant response [40] as they play an essential role in the defense against oxidative stress. Treatment with the crude extract and fraction F3 enhanced the activity of the antioxidant enzymes in HepG2 cells, as a protective mechanism to counter the increasing generation of ROS induced by H_2_O_2_. Previous studies reported that plant polyphenols including caffeic acid [41] and quercetin [42] induced the activities of SOD, GPx and CAT and strongly inhibited the generation of ROS in the oxidative stress-induced HepG2 cells, thus preventing or delaying conditions which favor oxidative stress in the cells.

In the present study, fraction F3 showed more pronounce effects than the crude extract in antioxidant potency including in enhancing antioxidant enzymes. The defense against ROS particularly in the liver is mediated by the transcription factor Nuclear factor E2-related factor 2 (NRF2). NRF2 activates and induces antioxidant responsive element (ARE) which results in the coordinated activation of genes that encode phase II detoxification and antioxidant enzyme expression [43]. Previous studies reported that phenolic acids including caffeic acid activated NRF2-ARE-mediated signaling in HepG2 cells by inducing the expression of genes encoding phase II drug metabolism/detoxification and anti-oxidative stress enzymes including SOD, CAT and GPx [44]. We speculated that bioactive compoundsin fraction F3 including caffeic acid could regulate the expression of antioxidant enzymes mediated by NRF2-ARE.

In this study, the multitude of polyphenolic compounds detected in the seed extracts of *T. indica* likely contributed to the high antioxidant activities. In fact, Siddhuraju (2007) had reported the potent antioxidant activities of the seed coat of *T. indica* [45]. Analyses for polyphenolic compounds in an earlier study reported the presence of mainly procyanidins in the seeds of *T. indica*, including the trimer, tetramer, hexamer and pentamer forms, procyanidin B2 and (-)-epicatechin [46]. Our analyses detected the additional presence of catechin, caffeic acid, ferrulic acid, chloramphenicol, myricetin, morin, quercetin, apigenin and kaempferol, which have not been previously reported. Procyanidin B2 is the major polyphenolic compound in the seed extract and it has several biological properties including antioxidant activity [47, 48] and anti-tumor effect [49]. As an antioxidant, procyanidin B2 can inhibit the generation of highly reactive species such as the hydroxyl radicals and chelates Fe (II) ions [50].

Fractionation of the methanol seed extract led to enhancement of their antioxidant potency as well as isolation of the bioactive compound. Previous studies have indicated the beneficial effect of fractionation in concentrating polyphenolic compounds, producing fractions with more potent antioxidant activities [51, 52]. UHPLC and NMR analyses of fraction F3 identified caffeic acid as the bioactive compound and this is the first report on its presence in *T. indica* seed. Caffeic acid, on its own can function as a potent antioxidant, and did not require the synergistic effects of the multitude of polyphenols present in the crude extracts. In addition to antioxidants, several biological activities have been attributed to caffeic acid including anti-inflammatory [52], anti-bacterial [53] and anti-carcinogenic effects [54]. The beneficial antioxidant properties of caffeic acid have been demonstrated in both *in vivo* and *in vitro* analyses involving various mechanisms including radical scavenging, metal ion chelation, and inhibitory actions on enzymes that induce free radical and lipid hydroperoxide formation [55]. The structural feature responsible for the antioxidant and radical scavenging activity of caffeic acid is the ortho-dihydroxyl group in the catechol ring. The presence of the electron-donating hydroxyl group at the ortho-position also lowers the O-H bond dissociation enthalpy and increases the rate of H-atom transfer to peroxyl radicals [56]. The unsaturated 2,3-double bond of the side chain also maximizes the stabilization of the polyphenolic radical [57].

## Conclusions

In this study, we identified the presence of ten polyphenolic compounds in the methanol seed extract of *T. indica* with seven of them reported here for the first time. Caffeic acid was identified as the most active compound in regards to antioxidant activities and was able to protect HepG2 cells against lipid peroxidation, possibly by acting as reducing agents, radical scavengers and by increasing activities of antioxidant enzymes. The seeds of *T. indica* have great potential for further research particularly in the area of oxidative stress and oxidative damage-related diseases. In addition, the high polyphenolic content and antioxidant activity of the seed makes them attractive ingredients in commercially-produced food-based products.
